# Antitumour potential of BPT: a dual inhibitor of cdk4 and tubulin polymerization

**DOI:** 10.1038/cddis.2015.96

**Published:** 2015-05-07

**Authors:** S Mahale, S B Bharate, S Manda, P Joshi, P R Jenkins, R A Vishwakarma, B Chaudhuri

**Affiliations:** 1 School of Pharmacy, De Montfort University, Leicester LE1 9BH, UK; 2 Medicinal Chemistry Division, CSIR-Indian Institute of Integrative Medicine, Canal Road, Jammu 180001, India; 3 Academy of Scientific and Innovative Research (AcSIR), CSIR-Indian Institute of Integrative Medicine, Canal Road, Jammu 180001, India; 4 Department of Chemistry, University of Leicester, Leicester LE1 7RH, UK

## Abstract

The marine natural product fascaplysin (**1**) is a potent Cdk4 (cyclin-dependent kinase 4)-specific inhibitor, but is toxic to all cell types possibly because of its DNA-intercalating properties. Through the design and synthesis of numerous fascaplysin analogues, we intended to identify inhibitors of cancer cell growth with good therapeutic window with respect to normal cells. Among various non-planar *tryptoline analogues* prepared, *N*-(biphenyl-2-yl) tryptoline (BPT, **6**) was identified as a potent inhibitor of cancer cell growth and free from DNA-binding properties owing to its non-planar structure. This compound was tested in over 60 protein kinase assays. It displayed inhibition of Cdk4-cyclin D1 enzyme *in vitro* far more potently than many other kinases including Cdk family members. Although it blocks growth of cancer cells deficient in the mitotic-spindle checkpoint at the G_0_/G_1_ phase of the cell cycle, the block occurs primarily at the G_2_/M phase. BPT inhibits tubulin polymerization *in vitro* and acts as an enhancer of tubulin depolymerization of paclitaxel-stabilized tubulin in live cells. Western blot analyses indicated that, in p53-positive cells, BPT upregulates the expression of p53, p21 and p27 proteins, whereas it downregulates the expression of cyclin B1 and Cdk1. BPT selectively kills SV40-transformed mouse embryonic hepatic cells and human fibroblasts rather than untransformed cells. BPT inhibited the growth of several human cancer cells with an IC_50_ <1 *μ*M. The pharmacokinetic study in BALB/c mice indicated good plasma exposure after intravenous administration. It was found to be efficacious at 1/10th the maximum-tolerated dose (1000 mg/kg) against human tumours derived from HCT-116 (colon) and NCI-H460 (lung) cells in SCID (severe-combined immunodeficient) mice models. BPT is a relatively better anticancer agent than fascaplysin with an unusual ability to block two overlapping yet crucial phases of the cell cycle, mitosis and G_0_/G_1_. Its ability to effectively halt tumour growth in human tumour-bearing mice would suggest that BPT has the potential to be a candidate for further clinical development.

A link between development of human cancers and cellular pathways where the retinoblastoma protein (pRb) has a major role is well established.^1, 2^ One of the frequent events associated with human tumour progression is abnormality in the pathway that links pRb, p16^INK4A^, cyclin D1 and Cdk4 (cyclin-dependent kinase 4).^3^ Cdk4 along with its activating cyclin partner D1 has a key role in cell cycle control.^4, 5^ The naturally occurring inhibitor of Cdk4-cyclin D1, p16^INK4a^ (p16), is a tumour supressor protein. Deletion or inactivating mutations in the p16 gene are observed in many human cancers.^6, 7^ The catalytic activity of Cdk4 depends on its activation by the protein cyclin D1, which is expressed during the G_0_/G_1_ phase of the cell cycle. Many cancers are characterised by abnormal overproduction of cyclin D1.^8, 9^ As Cdk4 inhibitors target a pathway that links pRb, p16INK4A, cyclin D1 and Cdk4, it makes inhibition of Cdk4-cyclin D1 enzyme a crucially important target for cancer chemotherapy.^10, 11, 12, 13^ However, Rb mutations, consistent with loss of Rb function, have been identified in a wide spectrum of tumours including osteosarcomas, small-cell lung carcinomas, breast carcinomas and others, and the Cdk4 inhibitors cannot inhibit such pathway involving Rb-mutated tumours.

A number of potential anticancer agents that selectively modulate the activity of Cdk4-cyclin D1 *in vitro* have been reported.^14^ These molecules also show the genotypic consequences of Cdk4 enzyme inhibition at the cellular level, that is, growth inhibition of cancer cells *in vitro*, arrest of asynchronous cells at G_0_/G_1_ and prevention of pRb phosphorylation at Cdk4-specific serine residues.^15, 16, 17^ Usually, competing with ATP molecules for binding at the protein kinase active site is the normal mechanism by which most small molecules inhibit kinase enzyme activity. Successful attempts to identify selective Cdk4 inhibitors using structure-based chemical design and molecular modelling have been reported.^18, 19, 20^ Furthermore, the success of Cdk4 inhibitors at clinical stages^21, 22, 23, 24, 25^ has indicated it as a promising therapeutic target for anticancer drug discovery.^14^

Fascaplysin (1), a natural product originally isolated from a marine sponge, specifically inhibits the Cdk4 enzyme.^26, 27^ It inhibits Cdk4-cyclin D1 with an IC_50_ of ~0.35 *μ*M and blocks growth of cancer cells at the G_0_/G_1_ phase of the cell cycle. Similar to cryptolepine and ellipticine,^28^ fascaplysin is also a planar structure and thus it intercalates double-stranded (d-s) DNA and shows unusual toxicity at the cellular level. It has been suggested that fascaplysin's planar structure is the possible explanation for its ability to intercalate d-s DNA and also its unusual toxicity at the cellular level. To overcome this unusual toxicity, recently we reported CA224 (2), a non-planar analogue of fascaplysin exhibiting selective Cdk4 inhibition with no DNA-intercalating property.^29^ In continuation to these efforts, herein we report identification of tryptoline-based compounds CA198 (3), CA199 (4), CA211 (5) and *N*-(biphenyl-2-yl)-tryptoline (BPT, 6) as selective Cdk4 inhibitors with no DNA-intercalating property. Based on the molecular modelling design, a number of non-planar analogues of fascaplysin were synthesized. They show specificity towards Cdk4-cyclin D1 enzyme activity and blocks the growth of cancer cells at the G_0_/G_1_ phase. Although BPT was also designed using a homology model of Cdk4, based on the X-ray crystallographic structures of Cdk2, Cdk4 and Cdk6,^30, 31, 32, 33^ further investigations showed that BPT blocks growth of cells at the G_2_/M phase, in a Cdk-independent manner, through inhibition of tubulin polymerization. BPT shows potent cytotoxicity in a panel of cancer cells and is efficacious against human tumours derived from HCT-116 and NCI-H460 cells in SCID mice models. Here, we present the biological activity of BPT in detail. BPT (6) was synthesized using a one-step procedure by coupling tryptoline with biphenyl 2-carboxylic acid. The chemical synthesis of BPT and chemical structures of 1-6 are shown in Figure 1.

## Results

### Selective inhibition of Cdk4-cyclin D1 by BPT

BPT inhibits Cdk4-cyclin D1 *in vitro* at low micromolar concentration (IC_50_=10 *μ*M) and is comparatively inactive against other members of Cdk family. BPT was tested against a panel of 58 representative kinases including Cdk5-p35, Cdk6-cyclin D1, Cdk7-cyclin H, EGFR, GSK3β, MAPK1, MEK1, PDGFR, Plk3, PKA, PKC*α*, IGF-1R, and so on, wherein it does not inhibit any kinase to any appreciable degree at 10 *μ*M (Table 1).

**Figure 1:**
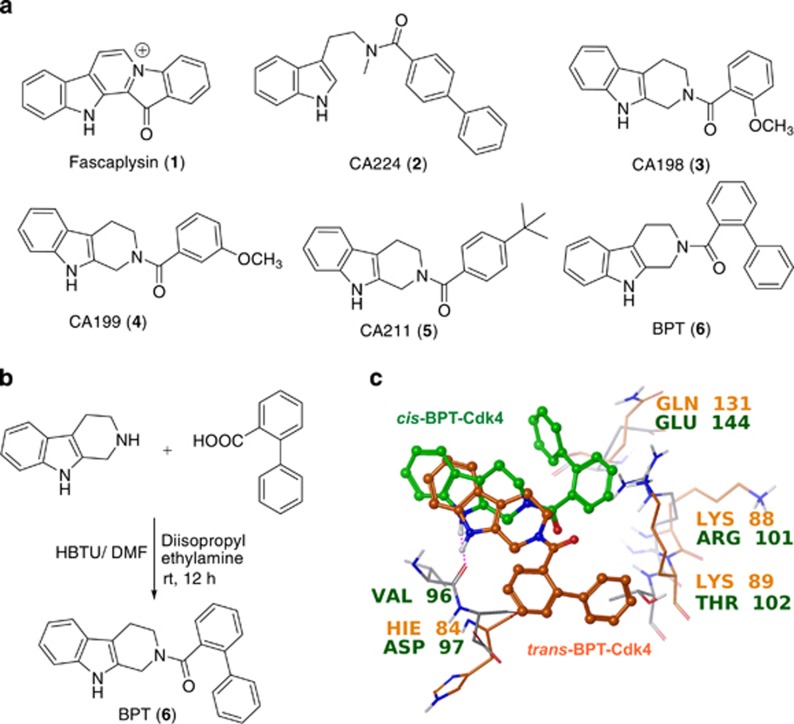
(**a**) Chemical structures of fascaplysin (**1**), CA224 (2) and its tryptoline analogues 3–6. (**b**) Synthetic scheme for BPT (**6**). (**c**) Molecular modelling studies to understand Cdk4 selectivity *versus* Cdk2: interactions of *cis*/*trans* conformations of BPT with Cdk2 and Cdk4, respectively (orange conformation is with Cdk2 and green with Cdk4)

To understand the observed selectivity towards Cdk4-cyclin D1 *versus* Cdk2-cyclin A, molecular modelling studies were performed.^33^ These two Cdks share 45% sequence homology; however, they differ by three peptidic sequences including 94–97 (Glu-His-Val-Asp)^Cdk4^/81–84 (Glu-Phe-Leu-His)^Cdk2^, 101–102 (Arg-Thr)^Cdk4^/88–89 (Lys-Lys)^Cdk2^ and Glu144^Cdk4^/Gln131^Cdk2^. BPT interacts with ATP-binding pocket of Cdk4-cyclin D1 with 83-fold selectivity with respect to Cdk2-cyclin A because of flexible conformational movement of the BPT amide bond, which allows free rotation of biphenyl ring leading to subsequent gain or loss of major hydrophobic interactions with one or other Cdk. BPT interacts with these Cdks in two different conformational states: (a) in *cis* conformation (green-coloured ligand in Figure 1c, *Ψ*=–9.6), it interacts selectively with the side chain of Arg101 residue of Cdk4-cyclin D1 by hydrophobic π–cation interaction, whereas in Cdk2-cyclin A, this interaction is missing as the corresponding Lys88 residue side chain orients away from BPT-binding cavity, and (b) in *trans* conformation (orange-coloured ligand in Figure 1c; *Ψ*=161.1), it interacts with Cdk2-cyclin A.

### DNA-binding assay

The ability of BPT to intercalate d-s DNA was important to segregate it from fascaplysin. It was studied by ethidium bromide (EtBr) dispacement assay and topoisomerase-I catalysed DNA relaxation or unwinding assay *in vitro*. Unlike fascaplysin, BPT failed to displace bound EtBr from DNA, indicating that it does not compete with EtBr-binding sites on DNA (EtBr is known to bind to the minor groove of d-s DNA, and also to DNA double helix and crosslinking sites^34^) and therefore shows no detectable affinity towards DNA (Table 1 and Supplementary Figure S5C). The interactions of fascaplysin, actinomycin D and BPT with pBluescript plasmid DNA is depicted in Supplementary Figure S5C. As indicated in Supplementary Figures S5A and C, 100 *μ*M of BPT was incapable of displacing 1.3 *μ*M of EtBr from pBluescript plasmid DNA. In contrast, DNA-intercalating agents actinomycin D and fascaplysin readily dislodges the bound EtBr. Usually, DNA intercalators hinder topoisomerase-catalysed DNA relaxation/unwinding process. Fascaplysin at low dose also hinder topoisomerase-I catalysed DNA relaxation, whereas BPT at much higher concentration than fascaplysin does not hinder DNA relaxation process, as indicated by two different bands on agarose gel (Supplementary Figure S5A). To insure that these results reflected a lack of DNA intercalation rather than an inhibition of topoisomerase-I enzyme activity, a second set of experiment was conducted using relaxed (i.e. negatively supercoiled) pBluescript plasmid DNA as initial substrate. BPT-treated negatively supercoiled DNA remained relaxed even after treatment with as high as 100 *μ*M concentration, whereas fascaplysin-treated negatively supercoiled DNA does not remain intact at low dose (1 *μ*M), clearly indicating the DNA-intercalating nature of the fascaplysin (Supplementary Figure S5B).

### Cancer cell growth inhibition

Cancer cell growth inhibition data for BPT (6) and its structural analogues 3–5 is presented in Table 2. These compounds were tested *in vitro* in 10 cancer cell lines known to be relatively resistant to known chemotherapeutic agents.^35^ The inhibitory effects of compounds were quantified using MTT assay. The results of cell proliferation assays indicate that BPT inhibits the growth of cancer cells *in vitro* at submicromolar concentrations. Among all the analogues, BPT was found to be the most potent compound at the cellular level.

### At its IC_50_ concentration, BPT arrests asynchronous cancer cells at the G_2_/M phase of the cell cycle (in p53^+^ A549 and p53^–^ NCI-H1299 cells)

**Figure 2:**
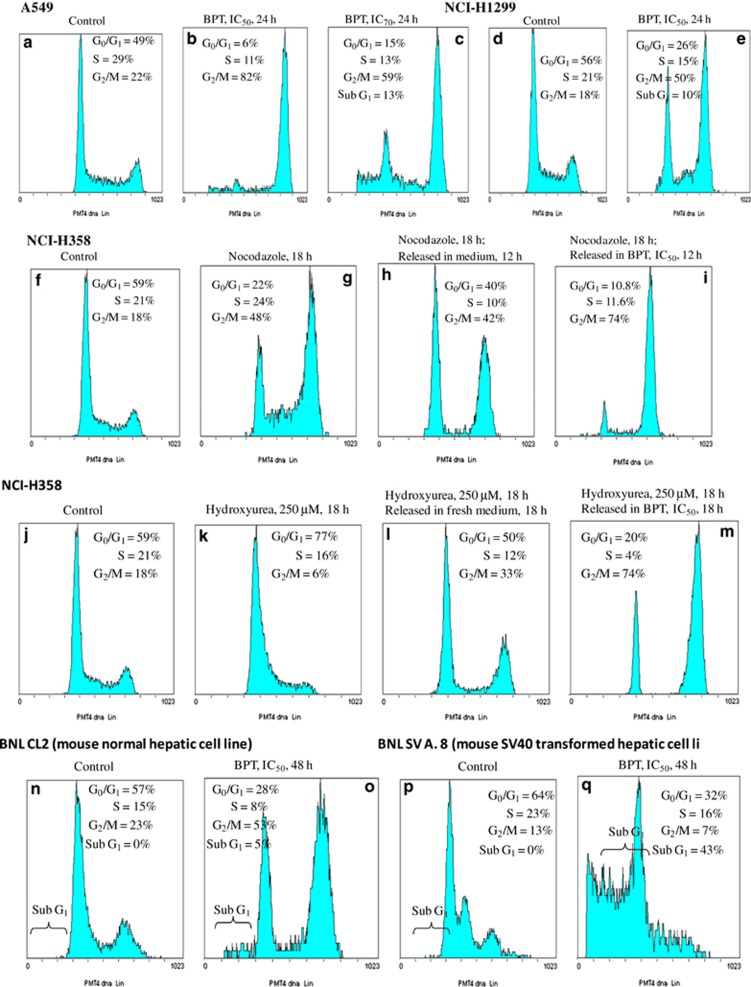
(**a**–**e**) Response of mitotic-spindle checkpoint-proficient human lung cancer cell lines, A549 and NCI-H1299, to BPT. Flow cytometric analysis of asynchronous cells show that majority of cells are arrested in the G_2_/M phase of cell cycle (4*n* DNA content) in both the cell lines. A549 untreated (**a**), treatment with IC_50_ concentration of BPT for 24 h (**b**) and treatment with IC_70_ concentration of BPT for 24 h (**c**); NCI-H1299 untreated or control (**d**) and treatment with IC_50_ concentration of BPT for 24 h (**e**). (**f–m**) Analysis of NCI-H358 cells using flow cytometer. Cells in the G_2_/M and G_1_/S phase synchronized by nocodazole and hydroxyurea, respectively, were released either in the fresh medium or in the fresh medium containing IC_50_ concentration of BPT, which exhibit greater tendency to block the cell growth at the G_2_/M phase. For nocodazole block experiment, figure show untreated or control cells (**f**), treated with 1 *μ*M nocodazole for 18 h (**g**), treated with 1 *μ*M nocodazole for 18 h and released in the fresh medium (**h**) and treated with 1 *μ*M nocodazole for 18 h and released in the presence of BPT, IC_50_ (**i**). For hydroxyurea block experiment, the figure shows untreated or control cells (**j**), treated with 250 *μ*M hydroxyurea for 18 h (**k**), treated with 250 *μ*M hydroxyurea for 18 h and released in the fresh medium (**l**) and treated with 250 *μ*M hydroxyurea for 18 h and released in the presence of BPT, IC_50_ (**m**). (**n**–**q**) Selective apoptosis in SV40-transformed cells by BPT was analysed by FACS. BNL CL2 (mouse embryonic normal hepatic cells) when exposed to BPT for 48 h at IC_50_ and IC_70_ concentrations show prominent G_2_/M arrest. As seen in the figure, untreated cells (**n**), treated with IC_50_ concentration of BPT, 48 h (**o**). BNL SV A.8 (mouse embryonic SV40-transformed hepatic cells) underwent apoptotic cell death upon incubation with BPT. The apoptosis was quantitated by measuring the % of cells that appeared in sub-G_1_ peak; 43 and 51% cells were found in sub-G_1_ peak after 48 h exposure with BPT, IC_50_ (**q**) concentration. The untreated cells (**p**) do not show any apoptosis

When A549 (p53+) cells were treated with BPT (IC_50_ concentration for 24 h), a profound block at the G_2_/M phase was observed with 82% cells appearing to be in the G_2_/M phase (Figure 2b). At the IC_70_ concentration, 59% of cells were blocked at the G_2_/M phase and 13% of cells appeared as apoptotic, whereas 15% of cells remained in the G_0_/G_1_ phase (Figure 2c). These results indicate that only at higher concentrations, BPT tends to act as a Cdk4 inhibitor blocking cells at the G_0_/G_1_ phase. At lower concentrations, greater tendency towards G_2_/M block is observed. Incubation of NCI-H1299 (p53-null) cells with the IC_50_ concentration of BPT resulted in a large number of cells (50%) accumulating at the G_2_/M phase (Figure 2e). A549 and NCI-H1299 cells, which are blocked profoundly at the G_2_/M phase by BPT, have a functional mitotic-spindle checkpoint. However in p53-null Calu-1 cells (with ‘non-functional' mitotic-spindle checkpoint), BPT, similar to compound 4,^30^ blocks only at the G_0_/G_1_ phase as a consequence of Cdk4-cyclin D1 enzyme inhibition (Supplementary Figure S4).

### BPT maintains nocodazole- and paclitaxel-induced G_2_/M block in NCI-H358 lung cancer cells

To induce a partial block at the G_2_/M phase so that cells are minimally stressed, p53-null NCI-H358 cells were treated with suboptimal concentrations of nocodazole (1 *μ*M) for 18 h. The blocked cells were released in the fresh medium when they readily entered the cell cycle without any apoptosis. In the presence of BPT, however, cells not only maintain the G_2_/M block but also >50% of the G_0_/G_1_- and S-phase cells enter the G_2_/M phase (Figures 2f–i) within 12 h. Although only representative histograms related to nocodazole treatment are shown, similar results were obtained when paclitaxel-blocked cells were released for 12 h in the presence of BPT.

### G_2_/M block of NCI-H358 cells by BPT, after release from hydroxyurea-mediated G_1_/S cell synchronization

Hydroxyurea is known to block cell growth at the G_1_/S boundary. Incubation with 250 *μ*M hydroxyurea for 18 h caused a block of NCI-H358 cells at the G_1_/S phase (77% cells were at the G_1_ phase; Figure 2g), at a stage of the cell cycle where Cdk2-specific inhibitors normally act. When released in the presence of BPT, cells proceed from the G_1_/S phase, confirming that BPT does not inhibit cellular Cdk2. Cells ultimately accumulate at the G_2_/M phase (72% Figure 2m). Cells released in the fresh medium enter the cell cycle (Figure 2k). These results again indicate that BPT has an inherent tendency to induce block at the G_2_/M phase.

### BPT selectively induces apoptotic cell death in SV40-large T-antigen-transformed normal mouse embryonic liver cells

SV40-large T-antigen inactivates both the tumour suppressor proteins p53 and pRb, and thereby transforms normal cells into tumorigenic cells. The effect of BPT on normal mouse embryonic hepatic (liver) cells BNL CL2 and its SV40-large T-antigen-transformed counterpart BNL SV A.8 was investigated. More than 50% normal cells appeared in the G_2_/M phase of the cell cycle upon 48 h incubation with IC_50_ and IC_70_ concentrations of BPT; however, only <10% cells were detected in the sub-G_1_ phase (Figure 2o). Interestingly, in the SV40-transformed cell line, significant apoptotic cell death was observed, which is represented by the percentage of cells appearing in the sub-G_1_ phase. After 48 h treatment with IC_50_ concentration of BPT, 43% cells were detected in sub-G_1_ (Figure 2q) phase. The percentage of apoptosis was increased to 51% at the IC_70_ concentration. Long-term cell survival assay in mouse embryonic normal hepatic cell line (BNL CL2) and SV40-mediated transformed mouse embryonic hepatic cell line (BNL SV A.8) after the treatment with BPT indicated that it selectively reduces the number of colonies in SV40-mediated transformed mouse embryonic hepatic cell line (BNL SV A.8) upon 12 days treatment but not in mouse embryonic normal hepatic cell line (BNL CL2) as shown in Supplementary Figure S3.

### Effect of BPT on the levels of cyclin B1, Cdk1, p53, p21^CIP1/WAF1^ (p21) and p27^KIP1^ (p27) in p53+ cells

**Figure 3:**
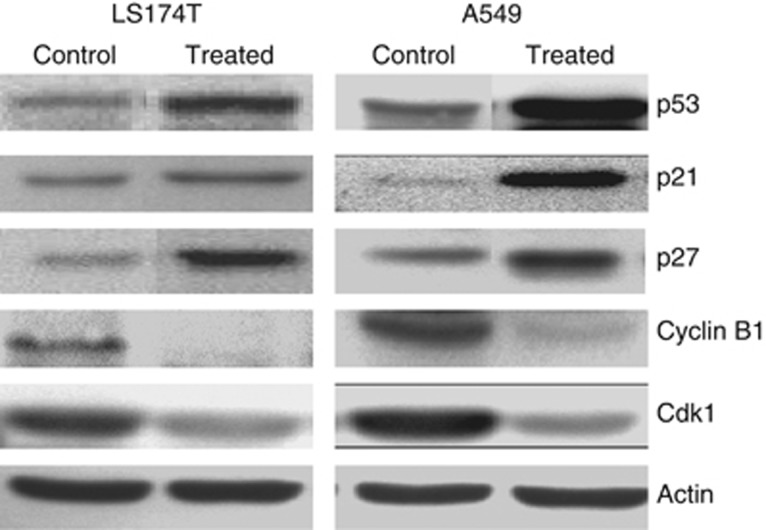
A549 and LS174T cells were analysed by western blot technique. The treatment of p53+ cells (A549 and LS174T) with BPT at IC_50_ concentration for 24 h resulted in induction of p53, p21^CIP1/WAF1^ (p21) and p27^KIP1^ (p27), whereas cyclin B1 and Cdk1 levels were downregulated

Western blot analyses of p53+ A549 and LS174T cells, after treatment with BPT at the IC_50_ concentration for 24 h, demonstrated more than 10-fold induction of p53, which corresponds with increase in levels of the Cdk global inhibitor p21^CIP1/WAF1^ (p21). The levels of the other pan-Cdk inhibitor p27^KIP1^ (p27) were also elevated owing to BPT treatment (Figure 3). The proteins Cdk1 and cyclin B1 were downregulated in the treated cells as compared with untreated control cells (Figure 3).

### Effect of BPT on the levels of cyclin B1, Cdk1, p53, p21 and p27 in mutant p53 cells

Western blot experiments were performed to ascertain the levels of cell cycle regulatory proteins in MIAPaCa cells, which contain p53 gene mutations (data not shown). Upon BPT treatment, p53, p21 and p27 levels remained unchanged, suggesting that the p21 and p27 induction is probably p53-dependent. The MiaPaca cell line has mutated p53 and proteins p21 and p27 are controlled by p53 pathway. The inhibition of cell growth by BPT was independent of the presence or absence of tumour suppressor protein p53, owing to which there was unchanged expression of these proteins after treatment with BPT. Interestingly, the cyclin B1 and Cdk1 levels were elevated and Tyr15 residue of Cdk1 remained dephosphorylated, indicating that Cdk1-cyclin B1 is still active in p53-mutated cells after BPT treatment.

In p53+ cancer cells, BPT downregulates cyclin B1 and Cdk1 but upregulates the pan-Cdk inhibitory proteins p21 and p27. This provides a mechanistic insight into the block of cell growth explaining why p53+ cells undergo block at the G_0_/G_1_, G_2_ and M phases of the cell division cycle. BPT upregulates cyclin B1 levels both in p53-mutant and p53-null cells (data not shown), indicating that in these cancer cells the major block occurs at a post-G_2_ phase of the cell cycle. This is likely to happen in between the G_2_ and the G_0_/G_1_ phases of the cell cycle, suggesting that BPT somehow affects the functions of mitosis.

### Inhibition of tubulin polymerization *in vitro* by BPT

**Figure 4:**
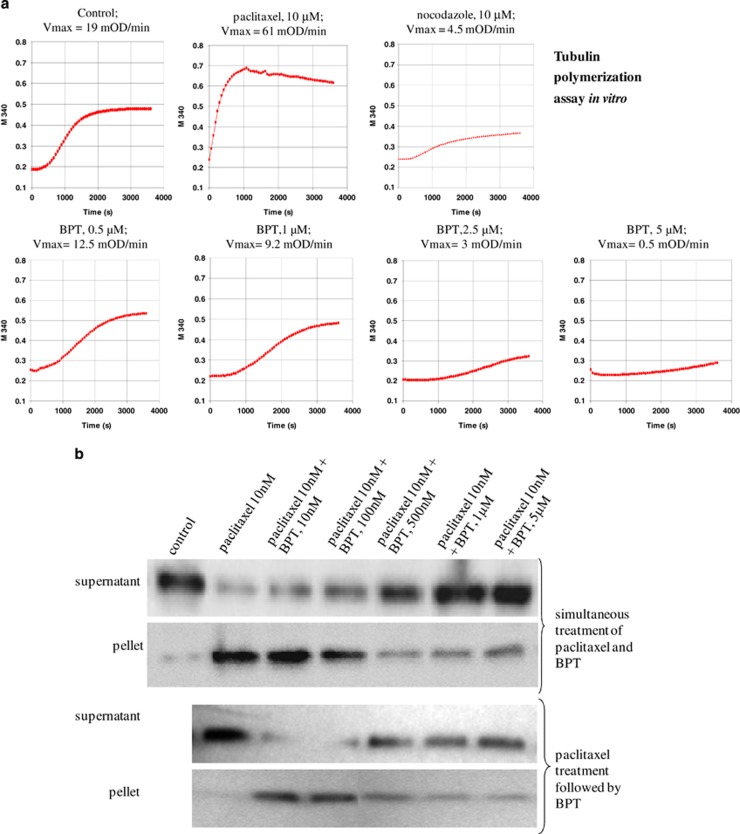
(**a**) Cell-free tubulin polymerization assay *in vitro*. Purified tubulin was used to test the ability of BPT to inhibit tubulin polymerization *in vitro*. The assay measures the increase in optical density as a result of tubulin assembly or polymerization. Nocodazole and paclitaxel were used in the assay as a known inhibitor and enhancer of tubulin polymerization. BPT was tested at four different concentrations that show inhibition of cell growth *in vitro*. The change in *V*_max_ value was used as an indicator of tubulin/ligand interactions. The polymerization curves indicate 0.5 , 1, 2.5 and 5 *μ*M of BPT reduced the *V*_max_ value from 19 mOD/min (control) to 12.5, 9.2, 3 and 0.5 mOD/min, respectively, in a dose-dependent manner. The curves shown represent the average of three independent experiments. (**b**) Inhibition of tubulin polymerization and enhancement of tubulin depolymerization in live cells. The tubulin polymerization assay was performed in A549 (whole cells) after 30 min compound treatment at the concentrations indicated in the figure. Supernatant and pellet represent unassembled and assembled tubulin, respectively. Tubulin polymerization is detectable by the increase of tubulin in pellet and its disappearance from supernatant. The western blots show dose-dependent inhibition of tubulin polymerization after the simultaneous treatment of paclitaxel and BPT that resulted in the accumulation of unassembled tubulin in supernatant. BPT also acts as an enhancer for tubulin depolymerization in a dose-dependent manner when paclitaxel-stabilized tubulin was subjected to BPT treatment for 30 min

BPT inhibits the polymerization of tubulin, which is concluded from the dose-dependent decrease in *V*_max_ (mOD/min) and reduction in final polymer mass (Figure 4a). When tested at four different concentrations, BPT decreased the *V*_max_ from 19 mOD/min to 12.5, 9.2, 3 and 0.5 mOD/min at 0.5, 1, 2.5 and 5 *μ*M of BPT, respectively. As a consequence of decreased *V*_max_, up to 80% reduction in final polymer mass was observed. Interactions of BPT with tubulin are shown in Supplementary Figure S1.

### Enhancement of tubulin depolymerization and inhibition of tubulin polymerization in the presence of BPT in live cells

A549 cells were used to explore the interactions between BPT and tubulin protein in live cells. As the mitotic-spindle checkpoint in A549 cells is intact, these cells are sensitive to antimicrotubule agents. The assembled (cytoskeletal) and unassembled (cytosolic) forms of tubulin were determined, via western blotting, from their accumulation and disappearance from pellet and supernatant fractions of the cell lysates, after cells were treated with BPT. Paclitaxel treatment of A549 cells for 30 min resulted in the accumulation of cytoskeletal tubulin as a consequence of enhanced tubulin polymerization, whereas in the presence of BPT the paclitaxel-mediated polymerization is inhibited in a dose-dependent manner (Figure 4b). More interestingly, when intracellular stabilized tubulin (paclitaxel-treated cells) was subjected to BPT treatment, BPT enhanced the tubulin depolymerization, resulting in the disappearance of cytoskeletal tubulin (pellet) form and accumulation of cytosolic tubulin (supernatant) form (Figure 4b).

### Clonogenic assay

**Figure 5:**
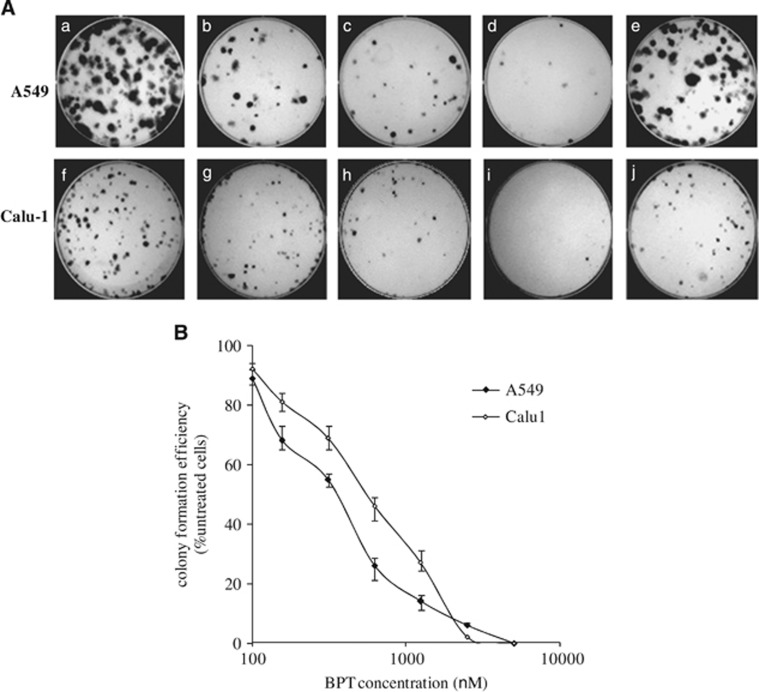
Long-term survival of cancer cells after the treatment with BPT. A549 and Calu-1 cells were investigated for their long-term survival efficiency after treatment with different concentrations of BPT. The colony formation efficiency is expressed as the percentage of colonies formed in the treated cultures compared with untreated cultures. (**A**) The representative plates show A549 untreated (a), treated with BPT, 0.5 *μ*M (b), treated with BPT, 1 *μ*M (c), treated with BPT, 2.5 *μ*M (d) and treated with fascaplysin, 0.8 *μ*M (e); Calu-1 untreated (f), treated with BPT, 0.5 *μ*M (g), treated with BPT, 1 *μ*M (h), treated with BPT, 2.5 *μ*M (i) and treated with fascaplysin, 1 *μ*M (j). (**B**) The curves representing colony formation efficiencies of A549 and Calu-1 cells with increasing concentrations of BPT. All results are represented by means±S.D. obtained from three independent experiments

The effects of BPT on colony formation efficiency of p53+ A549 and p53-null Calu-1 cells were investigated. Calu-1 cells, similar to A549, contain a functional pRb protein but, in contrast to A549 cells, have a defective mitotic-spindle checkpoint.^36^ The concentration at which BPT prevented colony formation (in a 12-day experiment) was found to be relatively lower than the concentration at which it inhibited cell proliferation (48 h experiment). This could be because very few cells in the total population retain the ability to maintain cell division cycles, which could eventually result in non-detectable colonies of cells. BPT shows significant reduction in the colony formation efficiency of both p53+ and p53-null cancer cells *in vitro* (Figure 5).

### *In vivo* experiments in mice: pharmacokinetics and determination of MTD

The pharmacokinetics of BPT was carried out in BALB/c mice at 10 mg/kg (*per os*) and 1.0 mg/kg (intravenous) dose, which showed AUC_0–*t*_ of 17.7 and 170 ng•h/ml, respectively. The PK parameters after intravenous dosing were: *t*_1/2_=0.19 h, *C*_max_: 413 ng/ml, C_0_: 888 ng/ml, AUC_0–*t*_=170 ng•h/ml, AUC_0–∞_: 174 ng•h/ml, CL: 95.8 ml/min per kg and *V*_d_: 1.56 l/kg. Because of the poor oral bioavailability of BPT, we decided to perform *in vivo* efficacy via intraperitoneal route. The *in vivo* study to determine maximum-tolerated dose (MTD) was performed in Swiss-albino mice over 2 weeks. Loss in animal body weight was considered as a measure of overtoxicity for the test compound. The concentration of the compound at which ≥10% weight loss was observed was determined and designated as MTD, although usually a weight loss, which is below 20% of the initial weight, is considered harmless as animals can recover once the treatment is stopped. The toxicity results obtained from these studies indicated that for BPT, the MTD in mice was ~1000 mpk (milligrams per kilogram of body weight).

### Effects on growth of tumours derived from HCT-116 and NCI-H460 cell lines

**Figure 6:**
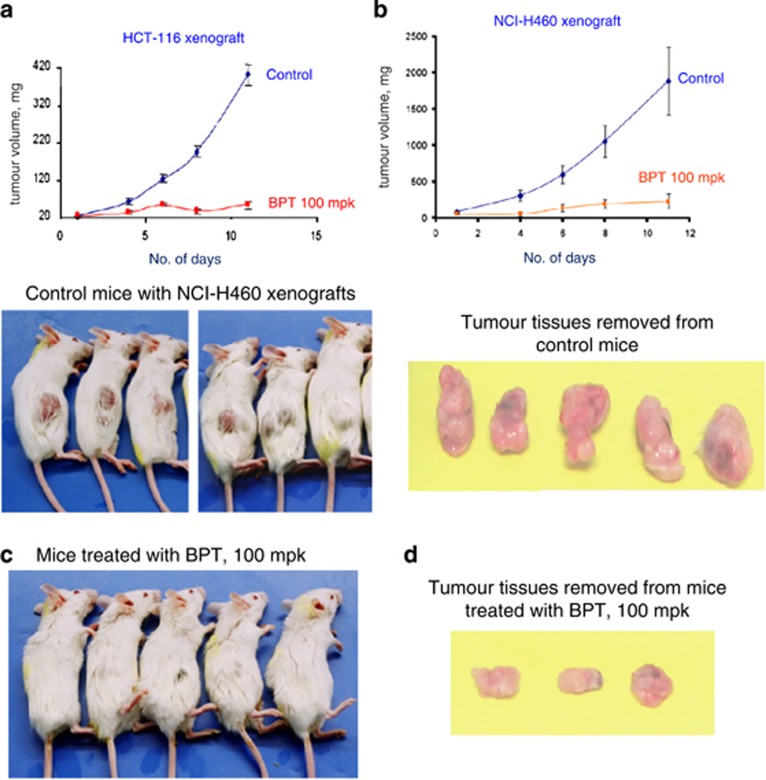
(**a**) *In vivo* tumour growth inhibition curve for BPT in the SCID mice-HCT-116 xenograft model. Graphs depict tumour growth inhibition in a group of animals treated with BPT at the concentration 100 mpk, which is compared with the untreated group of animals (shown in the graphs as the control group). Tumour sizes were recorded at 2–5 day intervals. Tumour weight (in mg) was estimated according to the formula for a prolate ellipsoid: (length (mm) × (width (mm)^2^) × 0.5) assuming specific gravity to be one and *π* to be three. (**b**) Tumour growth inhibition curves for BPT in the SCID mice-NCI-H460 xenograft model. Graphs depict tumour growth inhibition in a group of animals treated with BPT compared with the untreated group of animals (shown in the graphs as the control group). Tumour sizes were recorded at 2–5 day intervals. Tumour weight (in mg) was estimated according to the formula for a prolate ellipsoid: (length (mm) × (width (mm)^2^) × 0.5) assuming specific gravity to be 1 and *π* to be 3. (**c**) The pictures of SCID mice showing NCI-H460 tumour growth inhibition followed by treatment with BPT at the concentration 100 mpk. The treatments were continued for 9 consecutive days intraperitoneally when tumour growth had reached about 4–6 mm in diameter after about 6 days following the tumour cell injection. (**d**) Pictures of NCI-H460 tumour tissues, from SCID mice, exhibiting tumour growth inhibition by BPT

SCID mice, lacking both T and B immune cells, are an established model to study *in vivo* efficacy of potential anticancer agents. Flavopiridol (2.5 mpk) was used as positive control in both xenograft models (Supplementary Figure S8). When evaluated, BPT showed statistically significant (*P*<0.05) tumour growth inhibition (Figure 6a) at 1/10th of MTD concentration (100 mpk) in the HCT-116 tumour model. The data for BPT at 250 and 500 mpk is provided in Supplementary Figure S7. Mice injected with BPT exhibited ~80% tumour growth inhibition as compared with the untreated mice injected with the vehicle solution alone. A second set of SCID mice experiments, using tumours formed from NCI-H460 cells, confirmed the antitumour potential of BPT when it again showed high efficacy at 100 mpk concentration (Figures 6b–d). In both tumour models, treated animals displayed statistically insignificant weight loss (Supplementary Figure S6).

## Discussion

BPT, a non-planar analogue of fascaplysin, showed selective inhibition of Cdk4 with an IC_50_ of 10 *μ*M, showing no inhibition against other kinases including Cdks at 10 *μ*M. It does not intercalate DNA, which makes it free from unusual toxicity of DNA-intercalating agents. In cellular assays, BPT displays potent cytotoxicity in several cell lines. Considering the p53 and pRb status of the cancer lines tested, it is clear that inhibition of cell growth was p53- and pRb-independent. BPT showed cytotoxicity in p53-null (PC-3, Calu-1, NCI-H1299, NCI-H358) as well as p53-positive cells (LS174T, A549, NCI-H460). Furthermore, it also showed cytotoxicity in pRb-positive as well as pRb-null cells, indicating that inhibition of cell growth was independent of the presence or absence of tumour suppressor proteins p53 and pRb. High potency of BPT in cell lines that lack pRb activity (i.e. NCI-H358 that is pRb-null) suggests that Cdk4 inhibition may not be the only cellular target of BPT. It exhibits G2/M block of NCI-H358 cells, which again indicates that BPT has an inherent tendency to induce block at the G2/M phase. The cell cycle studies indicated that BPT blocks the G_0_/G_1_ phase of the cell division cycle as would be expected of a true Cdk4 inhibitor, but only partially. However, it profoundly blocks cells at the G_2_/M phase at comparatively low concentrations. G_2_/M arrest could be the result of cellular stress. In response to cellular stress, induction of the p53 protein may arrest cells at the G_2_/M phase.^37^ However, in p53-null NCI-H358 cells, the prometaphase block induced by nocadozole or paclitaxel is maintained by BPT. The G2/M block seems to be p53-independent. The selective cell death induction in cancer cells by BPT is very significant. From the results of cell cycle (Figures 2n–q), percent cell death and apoptosis (Supplementary Figure S2), and colony formation assay (Supplementary Figure S3), it was also observed that BPT selectively induces apoptosis in SV40-large T-antigen-transformed cells and not in -untransformed normal cells. A number of compounds, which are particularly potent Cdk2 inhibitors, have been reported to induce apoptosis selectively in transformed cells.^38, 39, 40^ Relatively low doses of celecoxib has been reported to induce G_2_/M arrest, followed by induction of apoptosis only in transformed cells but not in normal cells. Celecoxib also downregulates cyclin B1 and upregulates p21 expression independent of p53.^41^ In addition to manifesting these properties of celecoxib, BPT treatment also increases the level of p53 expression. Western blot studies indicate that repression of cyclin B1 and Cdk1 and elevated levels of p21 and p27 is a possible explanation of the G_2_/M block seen in p53 tumour suppressor-proficient A549 and LS174T cells (Figure 3).

Minor alteration of the microtubule dynamics can arrest the cell cycle progression at mitosis and eventually result in apoptotic cell death.^42, 43^ FACS analyses of cells treated with BPT indicate prometaphase block during the cell cycle. The growth of cancer cells *in vitro* is inhibited by BPT at much lower concentrations than it inhibits the enzyme Cdk4-cyclin D1 *in vitro*. In addition to these observations, the two- to three-fold resistance of BPT to inhibit growth of p53-null Calu-1 cells, in which the mitotic-spindle checkpoint involving tubulin is known to be abnormal,^44^ suggested that BPT may be an antimicrotubule agent. Therefore, the action of BPT on tubulin polymerization *in vitro* and in live cells was investigated (Figure 4a). BPT was found to inhibit polymerization and enhance depolymerization of tubulin. It is noteworthy to mention that previously discovered tubulin inhibitors are all relatively quite toxic in contrast to what we have discovered. As BPT shows dual mechanism of action at the cellular level, we explored the possibility of its potent cellular activity in reducing *in vivo* the long-term survival and colony formation efficiency of non-small-cell lung cancer (NSCLC) cells. BPT shows significant reduction in the colony formation efficiency of both p53+ and p53-null cancer cells. In *in vivo* efficacy studies, BPT showed significant antitumor activity at 1/10th of the MTD in HCT-116 and NCI-H460 xenograft models. In conclusion, a relatively non-toxic compound BPT with dual Cdk4/ tubulin polymerization inhibition activity and promising efficacy in *in vivo* tumour models has been identified.

## Materials and Methods

### Synthesis of BPT (6)

Dried round-bottomed flask (RBF) with stirrer bar was charged with biphenyl 2-carboxylic acid (1 mmol) and HBTU (1.1 mmol). Dry DMF (3 ml) was then added dropwise to the RBF. This was followed by the addition of tetrahydro-*β*-carboline (1.1 mmol) and diisopropyl ethylamine (1.1 mmol). The reaction mixture was then stirred for 12 h at room temperature. The reaction was extracted with ethyl acetate and cold water. The organic layer was separated, dried over anhydrous sodium sulphate and solvent was evaporated under reduced pressure. Crude product was purified by silica gel (nos. 100–200) column chromatography using EtOAc:hexane as mobile phase to get 2-(biphenyl-2-carbonyl)-2,3,4,9-tetrahydro-1*H*-pyrido[3,*4-b*]indole (BPT, 6) in 70% yield. White powder; yield: HPLC: *t*_R_=10.91 min (97% purity); ^1^H NMR (400 MHz, CDCl_3_, p.p.m.): *δ* (major rotamer) 8.29 (1H, s), 7.52–7.02 (13H, m), 5.12 (1H, d, *J*=16.0 Hz), 4.41 (1H, d, *J*=20 Hz), 3.49–3.43 (1H, m), 3.03–2.94 (1H, m), 2.62–2.50 (1H, m), 2.17–2.13 (1H, m); *δ* (distinct peaks for minor rotamer) 7.61 (1H, s), 4.20–4.11 (1H, m), 4.01–3.97 (1H, m), 3.91–3.87 (1H, m) 3.80–3.74 (1H, m), 2.78–2.74 (1H, m); ^13^C NMR (100 MHz, DMSO-*d*_6_, p.p.m.): *δ* (major rotamer) 169.02 (CO), 139.47 (C), 138.04 (C), 135.94 (C), 135.58 (C), 130.10 (C), 129.49 (CH), 128.49 (CH), 128.21 (CH), 128.03 (CH), 127.92 (CH), 127.68 (CH), 127.18 (CH), 126.25 (C), 120.78 (CH), 118.46 (CH), 117.43 (CH), 111.0 (CH), 106.35 (C), 44.54 (CH_2_), 39.62 (CH_2_), 20.85 (CH_2_); *δ* (distinct peaks for minor rotamer) 169.02 (CO), 139.11 (C), 138.31 (C), 135.84 (C), 135.64 (C), 130.0 (C), 129.42 (CH), 127.60 (CH), 127.35 (CH), 126.45 (C), 126.84 (CH), 117.47 (CH), 111.91 (CH), 107.03 (C), 44.40 (CH_2_), 39.52 (CH_2_), 20.24 (CH_2_); IR (KBr): *ν*_max_ 3396, 3275, 3057, 2959, 2924, 2850, 1720, 1630, 1612, 1481, 1442, 1426, 1381, 1354, 1306, 1281, 1232, 1160, 1135, 1074, 1040, 1018 cm^−1^; ESI-MS: *m/z* 353.10 [M+H]^+^; HR-ESI-MS: *m/z* 353.1681 calcd for C_24_H_21_N_2_O+H^+^ (353.1653).

### Kinase assays *in vitro*

The *in vitro* kinase assays for testing against Cdk4-cyclin D1, Cdk2-cyclin A, Cdk1-cyclin B1 and Cdk9-cyclin T1 were performed in-house. The methodology and results have been reported previously.^20, 30, 31, 32, 45^ The kinase profiling for 58 representative kinases was carrried out commercially at Millipore Bioscience Division, Livingston, UK.

### EtBr displacement assay

The assays were performed in a 96-well plate with clear bottom (Costar, Washington, DC, USA). The assay involved the addition of 10 *μ*l of 10 × concentrated stock solution of compounds (dissolved in DMSO and further diluted in EtBr displacement assay buffer) to 90 *μ*l of reaction mixture containing 6 *μ*g of purified pBlueScript DNA and 1.3 *μ*M EtBr in EtBr displacement assay buffer with final pH 7.4. Equivalent amounts of DMSO were added to the vehicle controls. In addition to control samples (DNA+EtBr), test samples (DNA+EtBr+test compounds), blank 1 (EtBr only), blank 2 (DNA only), wells containing DNA and compound were also prepared to test any change in the background fluorescence readings. The reduction in relative fluorescence counts was monitored (*λ*_excit_=260 nM; *λ*_emiss_=600 nM) and recorded after 1 min equilibration time. Fascaplysin and actinomycin D, which are known to intercalate d-s DNA, were used as standard compounds in assay. The mean control and test readings were corrected by subtracting blank readings. The percentage fluorescence in the test samples in reaction with control samples was calculated by using following formula:


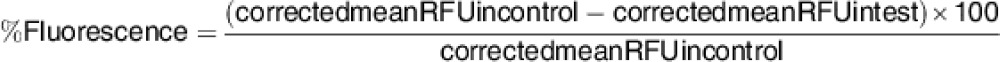


### Topoisomerase I catalysed DNA relaxation or unwinding assay *in vitro*

For assay, 5 nM supercoiled pBluescript d-s plasmid DNA and 10 U of topoisomerase-I active enzyme were used in each reaction well. To ensure that the assay determines the DNA-intercalating property of compounds and not topoisomerase I inhibition, in a parallel experiment relaxed plasmid DNA was first prepared by treating with topo I enzyme for 30 min and then used as an initial substrate for the assay. DNA relaxation assays were performed in the presence or absence of compounds in 40 *μ*l of DNA unwinding assay buffer. After 30 min incubation at 37 °C, reaction mixtures were treated with 3 *μ*l of 250 mM EDTA and extracted with phenol/chloroform. The DNA was dissolved in tris-EDTA buffer, pH 8.0. The samples (20 *μ*l) were treated with 2 *μ*l of 2.5% SDS, mixed with 2.5 *μ*l agarose gel loading buffer (10x) and subjected to electrophoresis on a 0.8% agarose gel without EtBr (separating the DNA in the presence of EtBr would convert the relaxed DNA into the supercoiled form). After the electrophoretic separation, DNA bands were stained with 1 *μ*g/ml EtBr and visualized using a UV illuminator. The compounds were compared with fascaplysin, which is a known DNA-intercalating molecule. Camptothecin, which is a known topoisomerase- I inhibitor, was used to test the activity and inhibition of enzyme.

### *In vitro* cell proliferation assays

All 10 human cancer cell lines were maintained at 37 °C in 5% CO_2_ in RPMI-1640 medium, supplemented with 10% foetal calf serum and 100 *μ*g/ml normocin. The 10 cancer cell lines used for screening were all obtained from ATCC (Manassas, VA, USA); they were the NSCLC (a form of cancer that is resistant to chemotherapy) lines: NCI-H460 (pRb^+^, p53^+^), A549 (pRb^+^, p53^+^), Calu-1 (pRb^+^, p53-null), NCI-H1299 (pRb^+^, p53-null), NCI-H358 (pRb-null, p53-null); the colon cancer line LS174T (pRb^+^, p53^+^); the prostate cancer line PC-3 (pRb^+^, p53-null); the pancreatic cancer line MiaPaca (pRb^+^, p53-mutant). The genotypes within brackets indicate the status of the tumour suppressor proteins pRb and p53. The mouse embryonic normal hepatic cell line (BNL CL2) and its SV40 large T-antigen-transformed counterpart cell line (BNL SV A.8) were also purchased from ATCC. The large T-antigen inactivates the tumour suppressor proteins p53 and pRb. The detailed procedure of cell proliferation (MTT) assay and IC_50_ determination was described previously.^32^

### Flow cytometric analysis

The untreated (control) and treated (with test compounds) cells were harvested by trypsinization, washed once with PBS and then fixed in 70% chilled (−20 ºC) ethanol for minimum 1 h. After the fixation step, cells were centrifuged for 5 min at 3000 × *g* at room temperature, and the pellet was suspended in PBS containing 50 *μ*g/ml propidium iodide (Sigma-Aldrich, Dorset, UK; cat. no. P-4170) and 0.5 mg/ml DNase-free ribonuclease (Sigma-Aldrich; cat. no. R-5503). The cells were stained for 1 h in the dark at 4 °C. Cell cycle analysis was performed on the Beckman Coulter (Epics Altra) fluorescence-activated cell sorter (Beckman Coulter UK Ltd, Buckinghamshire, UK). To gate all the events that represent single cells, and not cell doublets or cell clumps, the following analyses were performed on the samples. Cytograms of propidium iodide fluorescence peak signal *versus* integrated fluorescence or the linear signal were plotted. All data points on the straight line were isolated in a single gate and the gated data was further used for plotting a histogram that represents a complete cell cycle. The total number of events was not allowed to exceed 200 events per s. Data acquisition was stopped after a minimum of 10 000 events had been collected.

### Western blotting

A549, LS174T and MiaPaCa cells were seeded in 25 cm^2^ tissue culture flasks in the complete growth medium. When the culture flasks reached 40–50% confluency, cells were treated with BPT (IC_50_ concentration) for 24 h. Cells were harvested by trypsinization, washed in ice-cold PBS and then lysed in buffer (Sigma-Aldrich; cat. no. C-2978) containing protease inhibitor cocktail (Sigma-Aldrich; cat. no. P8340). The lysates were centrifuged at 14 000 r.p.m. (revolutions per minute) for 10 min at 4 °C and the total protein concentrations were estimated in the clear supernatants using the Bradford method. Equal amounts of protein (40 *μ*g) were loaded and electrophoresed on 10% SDS-polyacrylamide gels and blotted on Immobilon-P Transfer Membrane (Millipore; cat. no.IPVH20200). The blots were probed with respective primary antibodies (at 4 °C, overnight) at the following dilutions: cdc2 (New England Biolabs, Hitchin, UK; cat. no. 9110) at 1 : 1000 to detect Cdk1; cyclin B1 (CRUK, London, UK; cat. no. V152) at 1 : 1500 dilution to detect cyclin B1; Pab 1801 (Santa Cruz Biotechnology, Heidelberg, Germany; cat. no. sc-98) at 1 : 500 dilution to detect p53; N-20 (Santa Cruz Biotechnology; cat. no. sc-469) at 1 : 500 dilution to detect p21; C-19 (Santa Cruz Biotechnology; cat. no. sc-528) at 1 : 250 dilution to detect p27; AC-40 (Sigma-Aldrich; cat. no. A4700) at 1 : 2000 dilution to detect actin. Appropriate secondary antibodies conjugated with horseradish peroxidase were used and the protein bands were visualized by chemiluminescence using the ECL Kit (Santa Cruz Biotechnology; cat. no. sc-2048).

### Western blotting to test effect of BPT on tubulin polymerization and depolymerization of stabilized tubulin in cells

A549 (NSCLC) cells were seeded at a concentration of 10 000 cells per well in 1 ml complete growth medium in 24-well per 15 mm culture plates. The plates were incubated for 24 h for cell attachment and stabilization. In the first set of experiments, the cells were treated with 10 nM paclitaxel and different concentrations of BPT for 30 min (cells underwent simultaneous treatment of paclitaxel and BPT). In the second set of experiments, to study the effect of BPT on the stabilised form of tubulin, cells were pretreated with 10 nM paclitaxel for 30 min. The cell monolayer was washed two times with sterile PBS and fresh growth medium containing different concentrations of BPT were added. The plates were further incubated for 30 min, the cell monolayers were washed two times with sterile PBS at room temperature and then 100 μl tubulin extraction buffer (1 mM MgCl_2_, 2 mM EGTA, 0.5% NP40 and 20 mM Tris-HCl (pH 6.8)) supplemented with 2 mM phenylmethylsulfonyl fluoride, and a protease inhibitor cocktail (Sigma-Aldrich; cat. no. P8340) was added per well. After a short and vigorous vortex mixing, the cell lysates were incubated at room temperature for 5 min and then centrifuged at 16 000 r.p.m. for 10 min to separate the soluble and polymerized tubulin fractions. Each supernatant and pellet fraction was mixed with 10 × sample buffer, heated for 7 min at 95 °C and resolved on 10% SDS-polyacrylamide gels. The resolved proteins were then subjected to western blotting (as described above) with a specific α-tubulin antibody B-7 (Santa Cruz Biotechnology; cat. no. sc-5286).

### Cell-free tubulin polymerization assay *in vitro*

The purified tubulin was obtained commercially (Cytoskeleton Inc., Denver, CO, USA) and the polymerization assays were carried out according to the method described previously.^36^ Tubulin polymerization assay is based on the adaptation of the original methods of Lee and Timasheff,^46^ who demonstrated that light is scattered by microtubules to an extent that is proportional to the concentration of the microtubule polymer. The resulting polymerization curves are representative of the three phases of microtubule polymerization, namely nucleation, growth and steady-state equilibrium. Paclitaxel and nocodazole were used in the assay as a known enhancer and inhibitor of tubulin polymerization, respectively. The ability of BPT to inhibit tubulin polymerization *in vitro* was determined according to the manufacturer's instructions. Briefly, tubulin protein (3 mg/ml) was polymerized in GTP buffer (80 mM PIPES, pH 6.9, 2 mM MgCl_2_, 0.5 mM EGTA, 10.2% glycerol and 1 mM GTP) in the presence of a range of BPT concentrations at 37 °C in a temperature-regulated Biotech spectrophotometer (Potton, Bedfordshire, UK). The absorbance (at 340 nm) kinetics of 61 cycles for each sample was studied and the readings were recorded at an interval of 1 min.

### Colony formation assay

A549 and Calu-1 cells were plated at a concentration of 500 cells per well in 35 mm/6-well plates in 2 ml complete medium. The plates were incubated for 24 h stabilization and further incubated with a range of concentrations of fascaplysin and BPT for 24 h. Plates were then gently washed with PBS, replaced with fresh medium and incubated at 37 ºC. After 10–12 days, cells were fixed in methanol:acetic acid (2 : 1) fixative for 10 min, washed, air dried and stained with 1% crystal violet. The colonies were evaluated by visual counts. The number of colonies in treated cultures was expressed as a percentage of the control cultures. All results represent means and standard deviations from at least three independent experiments.

### Detection of apoptosis by nuclei staining with fluorescence dye DAPI

Mouse embryonic normal hepatic cell line (BNL CL2) and its SV40-mediated transformed cell line (BNL SV A.8) were used to study the levels of apoptosis. Apoptosis and nuclear fragmentation was detected using DAPI staining followed by observations under fluorescence microscope.^47^ Cells were seeded at a concentration of 50 000 cells per well in 35 mm/6-well plates in total of 2 ml complete growth medium. After 24 h stabilization period, cells were further incubated in the presence of IC_50_ concentration of BPT for 48 h. Followed by the drug exposure, the cells (along with the floating cells) were collected by trypsinization, washed in sterile PBS and fixed in ethanol:acetic acid (3 : 1) fixative for 10 min. The cell suspension was dropped on a glass slide to break open the cells and allowed to air dry. The smear formed on the slide was mounted in a medium containing 1 *μ*g/ml DAPI and covered with coverslip. The slides were observed under fluorescence microscope and minimum 500 nuclei were counted for each sample.

### Pharmacokinetic analysis

Oral and intravenous pharmacokinetic studies of BPT were carried out in BALB/c male mice of age 4–6 weeks, by administering BPT orally and intravenous formulation at dose of 10 mg/kg for oral and 1 mg/kg for intravenous. Plasma samples were collected at appropriate time points between the range 0 and 24 h, and analysed by LC-MS-MS. Mean plasma concentration calculated and data were further analysed to determine PK parameters such as *t*_1/2_, *C*_max_, ACU, *V*_d_ and CL, using WinNonlin 5.3 software package (Pharsight Company, Princeton, NJ, USA).

### MTD finding studies for *in vivo* experiments

Swiss-albino mice were used to determine the MTD for the compound. BPT was weighed and mixed with 0.5% (w/v) carboxymethylcellulose and triturated with Tween-20 (*secundum artum*) with gradual addition of water to make up the final concentration. Care was taken not to exceed >0.25% of Tween-20 in the final formulation of the BPT. In this study, six animals per group were administered with BPT at different doses for 5 days (Q1D × 5) *via* intraperitoneal route. Animals were monitored for weight loss, morbidity symptoms and mortality up to 2 weeks by the end of treatment. Significant weight loss was considered when mean animal weight dropped by ⩾10% and was considered highly significant when the drop was ⩾20%.

### Efficacy study in SCID mice

#### HCT-116 experiments

A group of 60 SCID (SCID strain-CBySmn.CB17-*Prkdc*^*scid*^/J; The Jackson Laboratory, Bar harbor, ME, USA; Stock no. 001803) male mice weighing 18–25 g and 6–8 weeks old were used for the studies. Human colon carcinoma, HCT-116 (ATCC; cat. no. CCL-247) cells were grown in McCoy's 5A medium supplemented with 10% FBS (Sigma-Aldrich). The cultured cells were injected subcutaneously into the dorsal side of SCID mice at the dose of 6.6 × 10^6^ cells in 0.2 ml of suspension. When the tumour growth reached to about 4–6 mm in diameter (over about 5 days), the animals were randomly divided into eight groups, each containing seven mice. The treatments were continued for 9 consecutive days intraperitoneally. Flavopiridol (2.5 mpk) was used as a positive control in this study.

#### NCI-H460 experiments

A group of 65 SCID (SCID strain-CBySmn.CB17-*Prkdc*^*scid*^/J; The Jackson Laboratory; Stock no. 001803) female mice weighing 15–24 g and 6–8 weeks old were used. Human non-small-cell lung carcinoma, NCI-H460 (ATCC; cat. no. HTB-177) cells grown in RPMI-1640 medium supplemented with 10% FBS (Sigma-Aldrich). The cultured cells were injected subcutaneously into the dorsal side of SCID mice at a tune of 5.3 × 10^6^ cells in 0.2 ml of suspension. When the tumour growth reached about 4–6 mm in diameter (about 6 days), the animals were randomly divided into eight groups, each containing 6 or 7 mice. The treatments were continued for 9 consecutive days intraperitoneally. Flavopiridol (2.5 mpk) was used as a positive control in this study.

#### Tumour weight measurements

Tumour size was recorded at 2–5 day intervals. Tumour weight (mg) was estimated according to the formula for a prolate ellipsoid: (length (mm) × width (mm)^2^) × 0.5) assuming specific gravity to be one and π to be 3. Tumour growth in compound treated animals is calculated as T/C (treated/control) × 100% and growth inhibition percent (% GI) was (100−% T/C).^48, 49, 50^

#### Body weight measurements

The body weights of animals in different treatment and control groups were monitored by taking the measurements daily during the treatment schedule. By considering the body weight at the start of the treatment as 100%, the percent weight loss was calculated on subsequent days of treatments.

#### Statistical analysis

Data from each experiment was analysed by Microsoft Excel 2000. Statistically significant differences were identified and analysed using Student's *t*-test for multiple comparisons *versus* control group.^48, 49, 50^ The experiments were performed by Piramal Life Sciences (Mumbai, India), on a service contract.

### Molecular docking and molecular dynamic simulations

The available crystal structures of Cdk4/cyclin D1 are in the apo form and have several missing residues, and thus they cannot be used for molecular modelling.^33^ In the present study, we have used a hybrid homology model of Cdk4/cyclin D1 described by Shafiq *et al.*,^51^ which was developed from the Cdk4/cyclin D apocrystal structure (PDB: 2W96) by incorporating positions of missing gaps and activation loops from Cdk2/cyclin A (PDB: 1FIN).^52^ This hybrid homology model was subjected to protein preparation wizard for H-bond optimization, heterogeneous state generation, protonation and overall minimization. Grid file of docking was constructed using XYZ coordinates of the N atom of Val96 residue with a binding site of 12 Å radius grid box (*X*=–10.521, *Y*=208.683, *Z*=107.944). For Cdk2 docking, the Cdk2 apoprotein (PDB ID: 1FIN) was subjected to protein preparation wizard for filling missing loops and side chains (using prime), ionization, H-bond optimization, heterogeneous state generation, protonation and overall minimization. Grid file of docking was constructed using XYZ coordinates of the N atom of Leu83 residue with a binding site of 12 Å radius grid box (*X*=–10.406, *Y*=209.105, *Z*=107.576).

For tubulin docking, the tubulin–colchicine complex (PDB ID: 1SA0) was retrieved from the protein data bank.^53^ In this complex, protein is heterodimeric in nature, consisting of two α-chains (451 residues), two *β*-chains (452 residues) and the Stathmin-like domain (142 residues). Crystal structure was subjected to protein preparation wizard for filling missing loops and side chains (using prime), ionization, H-bond optimization, heterogeneous state generation, protonation and overall minimization. All other ligands, water and ions were removed except colchicine. Grid file for docking was constructed considering colchicine ligand as centroid of grid box of 10 Å size at interphase of *α/β* tubulin (C and D chains). All ligands were sketched in Maestro, prepared using ligprep and docked by Glide molecular docking software (Schrodinger LLC, Bangalore, India) in XP mode.

The Cdk4-BPT docked complex obtained from XP docking was subjected to system builder, in which TIP4P-Ew was used as an aqueous solvent model. The cubic box of 12 Å radius was used to define the core and overall complex was neutralized by adding one Cl^−^ counter ion for simulation. Further this complex was minimized by steepest descent method followed by the Broyden–Fletcher–Goldfarb–Shanno algorithm with convergence threshold of 2.0 kcal/mol and overall 1000 iterations. MD simulations were carried out at normal temperature and pressure (300 °K and 1.01325 bar, respectively). Thermostat and barostat method opted was langevin with ensemble pathway comprising NVT (constant number of particles, volume and temperature) and isotropic coupling method. Overall model system was relaxed before 10 ns simulation and coulombic interactions were defined by short-range cutoff radius of 9.0 Å and by long-range smooth particle mesh Ewald tolerance to 1e−09.

## Figures and Tables

**Table 1:** Activity of fascaplysin, BPT and other structural analogues in different *in vitro* kinase assays and DNA-binding (EtBr displacement) assay

***In vitro* bioassay**	**IC_50_ (mean±S.D.) (*μ*M)**				
	**CA198 (3)**	**CA199 (4)**	**CA211 (5)**	**BPT (6)**	**Fascaplysin (1)**
Cdk4-cyclin D1	25±5.5	24.4±4	44±3	10±1.2	0.41±0.04
Cdk2-cyclin A	861±29	766±33	720±24	831±15.5	>250
Cdk1-cyclin B1	>500	>500	>500	>500	>250
Cdk9-cyclin T1	>1000	>1000	>1000	>1000	>250
Cdk5-p35	ND	ND	ND	NI	ND
Cdk6-cyclin D1	ND	ND	ND	NI	ND
Cdk7-cyclin H	ND	ND	ND	NI	ND
EGFR	ND	ND	ND	NI	ND
GSK3*β*	ND	ND	ND	NI	ND
MAPK1	ND	ND	ND	NI	ND
MEK1	ND	ND	ND	NI	ND
PDGFR	ND	ND	ND	NI	ND
Plk3	ND	ND	ND	NI	ND
PKA	ND	ND	ND	NI	ND
PKC*α*	ND	ND	ND	NI	ND
IGF-1 R	ND	ND	ND	NI	ND
EtBr displacement assay	No EtBr displacement up to 100 *μ*M	No EtBr displacement up to 100 *μ*M	No EtBr displacement up to 100 *μ*M	No EtBr displacement up to 100 *μ*M^a^	Displaces EtBr at 1 *μ*M

Abbreviations: ND, not determined; NI, no inhibition up to 10 *μ*M

IC_50_ values are presented in *μ*M concentration. The IC_50_ values presented here are means and S.D. from three independent experiments

a Detailed results of DNA-binding studies are provided in Section S5 of the Supplementary Information

**Table 2:** IC_50_ concentrations expressed in *μ*M for the *in vitro* cell growth inhibition induced by exposure to fascaplysin (1), CA198 (3), CA199 (4), CA211 (5) and BPT (6) for 48 h and measured by MTT assay

**Cell lines**	**Fascaplysin (1)**	**CA198 (3)**	**CA199 (4)**	**CA211 (5)**	**BPT (6)**
LS174T (colorectal carcinoma; p53+, pRb+)	0.88+0.04	33+2.5	7.1+1.3	29+2	0.85+0.07
PC-3 (prostate; p53 null, pRb+)	0.92+0.06	42+3	13.1+1.8	32+1.5	0.74+0.09
MiaPaCa (pancreatic; p53His273mut, pRb+)	0.88+0.2	43+5	8.5+ 1.3	29+4.5	0.8+0.1
A549 (NSCLC; p53+, pRb+)	0.69+0.03	46+4	8.0+2	31+3.5	0.92+0.08
Calu-1 (NSCLC; p53 null, pRb+)	1.3+0.1	92+4.5	47+3.5	54+4	2.8+0.4
NCI-H460 (NSCLC; p53+, pRb+)	ND	29+3.5	6.2+0.7	22+2	0.6+0.06
NCI-H1299 (NSCLC; p53 null, pRb+)	ND	35+2	4.5+0.5	30+1	0.9+0.045
NCI-H358 (NSCLC; p53 null, pRb null)	ND	28+4	3.5+0.8	25+1.5	0.68+0.07
BNL CL2 (mouse normal hepatic cells)	ND	37+1.5	6.5+0.7	27+2.5	0.72+0.1
BNL SV A.8 (mouse hepatic; SV40-mediated transformed cells)	ND	40+3.5	8.2+1	35+3	0.75+0.08

All results represent means and S.D. from at least three independent experiments

## Abbreviations

Cdk: cyclin-dependent kinase
d/w: distilled water
d-s: double-stranded
EtBr: ethidium bromide
mpk: milligrams per kilogram of body weight
MTD: maximum-tolerated dose
NSCLC: non-small-cell lung cancer
pRb: retinoblastoma protein
r.p.m.: revolutions per minute
SCID: severe combined immunodeficient
