# Supplementary material for: Antitumour potential of BPT: a dual inhibitor of cdk4 and tubulin polymerization
Source: Cell Death Dis. 2015 May 7;6(5):e1743–. doi: 10.1038/cddis.2015.96 (PMC4669722; doi:10.1038/cddis.2015.96)
Supplement: Supplementary Information [file cddis201596x1.doc]

**Supporting Information**

**Anti-tumour Potential of N-(Biphenyl-2yl)-Tryptoline (BPT): A Dual Inhibitor of Cdk4 and Tubulin Polymerization**

Sachin Mahale^1^, Sandip B. Bharate^2,3^,*, Sudhakar Manda^2,3^, Prashant Joshi^2,3^, Paul R. Jenkins^4^, Ram A. Vishwakarma^2,3^, Bhabatosh Chaudhuri^1*^

^1^School of Pharmacy, De Montfort University, Leicester, LE1 9BH, UK

^2^Medicinal Chemistry Division, CSIR-Indian Institute of Integrative Medicine, Canal Road, Jammu-180001, India

^3^Academy of Scientific & Innovative Research (AcSIR), CSIR-Indian Institute of Integrative Medicine, Canal Road, Jammu-180001, India

^4^Department of Chemistry, University of Leicester, Leicester, LE1 7RH, UK

*Corresponding author: Professor B. Chaudhuri, School of Pharmacy, De Montfort University, Leicester, LE1 9BH, UK; Fax: +44(0) 116 257 7287; Tel: 44(0)116 250 7280; E-mail: [*bchaudhuri@dmu.ac.uk*](mailto:bchaudhuri@dmu.ac.uk) (BC), [*sbharate@iiim.ac.in*](mailto:sbharate@iiim.ac.in) (SBB).

**Contents**

**S1**. Molecular modeling of BPT with tubulin

**S2**. Selective killing of SV40 transformed mouse embryonic hepatic cells by BPT

**S3**. Long term survival of mouse embryonic normal hepatic cell line (BNL CL2) and SV40 mediated transformed mouse embryonic hepatic cell line (BNL SV A.8) after the treatment with BPT.

**S4**. Cell cycle analysis of Calu-1 cells upon BPT treatment

**S5.** DNA binding studies of BPT and fascaplysin

**S6**. Effect of BPT on body weight of the mice in the SCID mice-HCT116 xenograft model

**S7.** Effect of BPT in xenograft models at 250 and 500 mpk

**S8.** Effect of flavopiridol in HCT-116 xenograft model

**S9.** NMR, MS and HPLC data Scans of BPT

**S1. Molecular modeling of BPT with tubulin**

Microtubules, the key components of cytoskeleton, are made up of α/β-tubulin heterodimers. Microtubule assembly has been targeted using number of polymerization inhibitors and inducers, by binding at different sites including a) colchicine binding site; at interphase of the α/β tubulin heterodimer and b) taxol and vinblastine binding site, deep inside β-tubulin. The mechanism of tubulin polymerization inhibitor involves binding at the interphase of the α/β tubulin and forming complex with tubulin like colchicine. This complex is added to the microtubule assembly, where it induces unfavourable conformational changes in tubulin dimer (M-loop) and thus further polymerization process gets stopped. Furthermore, tubulin-polymerization inhibitor complex perturbs microtubule growth by sterically blocking further addition of the tubulin dimers to form microtubule assembly. Upon molecular docking studies, it was observed that BPT binds at α/β-tubulin interphase by H-bonding with residue Thr179. In addition to this, hydrophobic biphenyl ring fits in the hydrophobic core of the β-tubulin formed by Leu248, Ala250, Leu252, Cys241, Leu255, Ala316, Ala317 and Ala354 (Fig.16b) . Interestingly, these interactions were missing in fascaplysin. BPT binding at interphase of α/β-tubulin, is supposed to induce conformational changes in protein which further perturbs tubulin polymerization to form microtubule assembly.


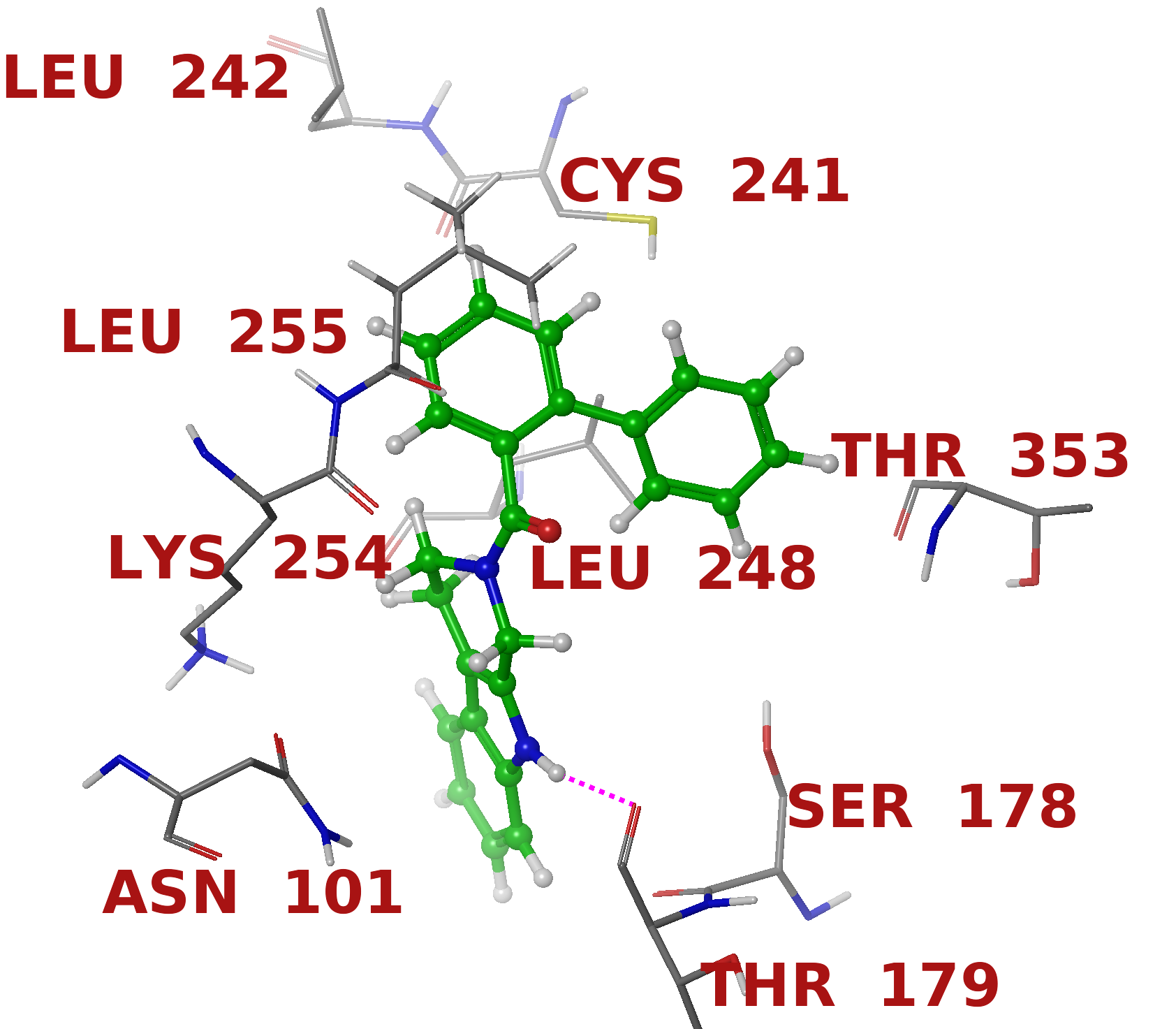


**Fig. S1.** BPT interactions at α/β-tubulin interphase.

**S2.** **Selective killing of SV40 transformed mouse embryonic hepatic cells** **by BPT**

**Fig. S2.** **Selective killing of SV40 transformed mouse embryonic hepatic cells.** BNL CL2 (normal) and BNL SV A. 8 (SV40 transformed) cells upon incubation with BPT for 48 h indicate selective apoptosis in SV40 transformed cells as compaired with the normal counterpart. (A) The graphs show percent cell death measured by trypan blue dye exclusion method in two cell lines (BNL CL2 and BNL SV A.8) after treatment for 48 h with increasing concentrations of BPT. The graphs also show cell growth inhibition measured by MTT assay in BNL CL2 and BNL SV A. 8 cells after BPT treatement for 48 h. (B) DAPI stained, fluorescence microscopic pictures captured at 40X magnification. BNL SV A.8 untreated cells (a), treated with BPT (IC_50_) for 48 h (b), BNL CL2 untreated cells (c), treated with BPT (IC_50_).for 48 h. Minimum 500 nuclei were counted for each sample. The fragmented nuclei and apoptotic cells are indicated with arrows.

**S3.** **Long term survival of mouse embryonic normal hepatic cell line (BNL CL2) and SV40 mediated transformed mouse embryonic hepatic cell line (BNL SV A.8) after the treatment with BPT**

**Fig. S3:** **Long term survival of Mouse embryonic normal hepatic cell line (BNL CL2) and SV40 mediated transformed mouse embryonic hepatic cell line (BNL SV A.8) after the treatment with BPT.** The cells were treated with the diffrent concentrations of BPT and then incubated in drug free medium for 12 days. the colonies were fixed in methanol : acetic acid (2: 1) and stained with 1% crystal violet and representative plates were scanned using the gel documentation system . The figure shows BNL CL2 cells (a) untreated cells, (b) treated with DE002, IC_20_ (0.25 µm), (c) treated with DE002, IC_30_ (0.4 µm) (d) treated with DE002, IC_50_ (0.6 µm) and (e) treated with DE002, IC_70_ (0.9 µm) and BNL SV A.8 cells (f) untreated cells, (g) treated with DE002, IC_20_ (0.25 µm), (h) treated with DE002, IC_30_ (0.4 µm) (i) treated with DE002, IC_50_ (0.6 µm) and (j) treated with DE002, IC_70_ (0.9 µm).

**S4.** **Cell cycle analysis of calu-1 cells upon BPT treatment**

**A B**

**Fig. S4.**  **Cell cycle analysis of calu-1 cells upon BPT treatment.** FACS analysis of serum-starved Calu-1 cells released in the presence of BPT and Western blot analysis of proteins from asynchronous Calu-1 cells treated with BPT. The results support the possible inhibition of the Cdk4-cyclin D1 enzyme at the cellular level in the mitotic spindle checkpoint-deficient Calu-1 cells after treatment with BPT. Figure S4.A shows FACS analyses of untreated or control cells (A), serum-starved cells for 24 h (B), serum-starved cells released in the presence of BPT at the IC_50_ concentration for 24 h (C), and serum-starved cells released in the presence of BPT at the IC_70_ concentration for 24 h (D). Two to three-fold increase in the G_1_:S ratio was considered as an indication of the G_0_/G_1_ arrest of cells. Figure S4B shows on Western blots the status of pRb phosphorylation in Cdk4-specific serine residues after a 24 h treatment of asynchronously growing Calu-1 cell with BPT. The phospho-specific polyclonal antibodies Ser780, Ser795 and Ser807/811 detect pRb phosphorylated at Serine residues 780, 795 and 807/811 respectively. The monoclonal antibody pRb (4H) detects the total phosphorylated and unphosphorylated pRb protein. Figure 4B shows proteins from untreated cells (C), from cells treated with IC_50_ concentration of fascaplysin, 24 h (T1), from cells treated with IC_50_ concentration of BPT, 24 h (T2), and from cells treated with IC_70_ concentration of BPT, 24 h (T3).

**S5. DNA binding studies of BPT and fascaplysin**


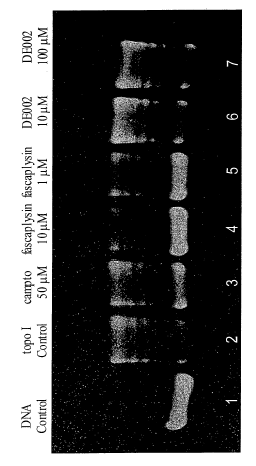


**Figure S5(A): BPT does not intercalate with pBlueScript plasmid DNA.** The ability of BPT to intercalate DNA was investigated using a topoisomerase I catalysed DNA unwinding assay and compared with the results obtained using Fascaplysin and DNA-intercalating agent, Camptothecin. The unwinding/relaxation assay were carried out as described. The final products of topoisomerase I relaxation assay were resolved on o.8 % agarose gel and were stained with 0.5 mg/ml ethidium bromide in order to visualise with UV illumination. Lane 1 contained the control pBlueScript DNA showing the super coiled form. Lane 2 contained the control relaxation reaction with topoisomerase I enzyme without any compound. Lane 3, 4,5,6 and 7 contained the topoisomerase I relaxation reaction carried in the presence of camptothecin (50 µM), fascaplysin (10 and 1 µM) and BPT (10 and 100 µM).


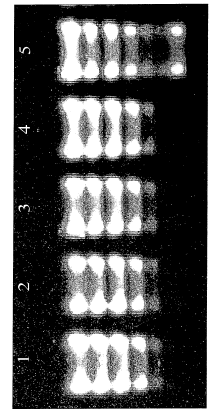


**Figure S5(B) : Topoisomerase I treated plasmid DNA was further subjected to BPT treatment.** The reaction products were resolved on a 0.8 % agarose gel stained with 0.5 mg/ml ethidium bromide in order to visualize DNA with UV illumination. The figure shows relaxed pBlueScript plasmid DNA (lane 1), relaxed plasmid DNA treated with 1 µM, 10 µM and 100 µM of BPT (lane 2,3 and 4 respectively) and relaxed plasmid DNA treated with fascaplysin 1 µM (lane 5).


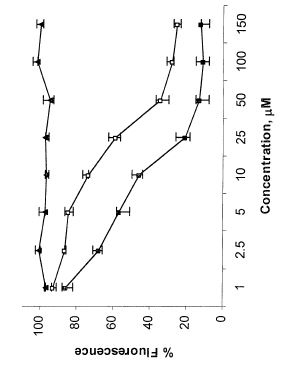


**Figure S5(C): BPT does not displace ethidium bromide from the minor groove of double-stranded DNA molecule.** The ability of fascaplysin, BPT (a non-planar analogue of fascaplysin) and actinomycin (a known DNA intercalator) to interact with the minor groove of DNA was determined by the fluorescence-based ethidium bromide displacement assay. The final concentration of ethidium bromide in the assay was 1.3 µM. The figure shows representative curves with increasing concentrations of fascaplysin (filled squares), actinomycin D (unfilled squares) and BPT (filled triangles). The result represents means and standard deviations from three independent experiments.

**S6. Effect of BPT on body weight of the mice in the SCID mice-HCT116 xenograft model**

The weight loss observed in treated animals was found to be <10% of the starting weights of the animals (Fig. S5) in the HCT116 tumour model. This loss of weight can be considered to be statistically insignificant indicating that compound treatment caused no major toxicity or harm to the animals. The body weight of the animals at the beginning of treatment was measured and this was considered to be 100%. The percentage weight loss or gain was calculated using the initial weight as a reference. Similar results were obtained in NCI-H460 tumour model (data not shown). BPT shows minimal toxicity in animal models and can be tolerated up to 1000 mpk concentration without any significant toxicity.

**Fig. S6.** **Effect on body weight of the mice in the SCID mice-HCT116 xenograft model**. The body weights of untreated and treated with BPT (100 mpk) animals were monitored by taking measurements daily during the treatment schedule. By considering the body weight at the start of treatment as 100%, the percentage weight loss was calculated on subsequent days of treatment.

**S7. Effect of BPT in xenograft models at 250 and 500 mpk**

**Figure S7.** Tumor growth inhibition curve for BPT (at 250 and 500 mpk) in in-vivo HCT116 xenograft model

**S8. Effect of flavopiridol in HCT116 xenograft model**

**Figure S8.** Tumor growth inhibition curve for flavopiridol in in-vivo HCT116 xenograft model.

**S9. NMR, MS and HPLC Data Scans of BPT**

**HPLC data of BPT**

**HPLC conditions**: HPLC analysis was done on Shimadzu HPLC system (model: LC-6AD) equipped with a PDA detector (model: SPD-M20A) using Inertsil C8 (3.5 μ, 4.6× 250 mm) column. Mobile phase used was ACN: water gradient elution (ACN: 50% at 0 min, 70% at 10 min, and 90% at 15-30 min) at flow rate of 1 ml/min (run time = 30 min).

**Chromatogram:**
